# Racial disparities of differentiated thyroid carcinoma: clinical behavior, treatments, and long-term outcomes

**DOI:** 10.1186/s12957-018-1340-7

**Published:** 2018-03-05

**Authors:** Jianing Tang, Deguang Kong, Qiuxia Cui, Kun Wang, Dan Zhang, Xing Liao, Yan Gong, Gaosong Wu

**Affiliations:** 1 Department of Thyroid and Breast Surgery, Zhongnan Hospital of Wuhan University, 169 Donghu Road, Wuhan, Hubei 430071 China; 2 Department of General Surgery, Zhongnan Hospital of Wuhan University, Wuhan, Hubei China; 3 Department of Thyroid and Breast Surgery, Tongji Hospital, Huazhong University of Science and Technology, Wuhan, Hubei China; 4 Department of Biological Repositories, Zhongnan Hospital of Wuhan University, 169 Donghu Road, Wuhan, Hubei 430071 China

**Keywords:** Differentiated thyroid carcinoma, Race, SEER

## Abstract

**Background:**

The incidence of thyroid cancer in black Americans is significantly lower than that in white Americans, and the impact of race on the prognosis of thyroid cancer remains controversial. The purpose of this study was to determine the risk factors for survival in black and white patients and to compare the survival of differentiated thyroid carcinoma subtypes between these two races. We further investigated the association of lymph node and distant metastases with races.

**Methods:**

This is a retrospective analysis using data from the National Cancer Institute’s Surveillance, Epidemiology, and End Results Program. A total of 70,346 cases were included in our study. Patients’ demographics and cancer- and treatment-related characteristics were compared between the black and white Americans using chi-square and Fisher’s exact tests. For multivariate analysis, Cox proportional hazards regression were used to assess the association between potential risk factors and the survival in black and white patients.

**Result:**

Black Americans had a worse overall survival than white Americans (HR = 1.127, *P* = 0.002). While disease-specific survival **(**DSS) was comparable, the risk factors for DSS were different between white and black Americans. Black Americans had less lymph node metastasis of classical variant papillary thyroid carcinoma (CPTC, OR = 0.476, *P* < 0.001) and follicular variant papillary thyroid carcinoma (FVPTC, OR = 0.522, *P* < 0.001), but not follicular thyroid carcinoma (FTC). However, black Americans with FVPTC, but not CPTC or FTC, had a higher potential of distant metastasis (OR = 1.715, *P* = 0.026). Furthermore, only white patients with tumor > 2 cm and lymph node metastasis benefited from radioactive iodine.

**Conclusions:**

The risk factors for DSS were significantly different in white and black patients. The impact of race should be considered in treatment strategy for thyroid cancer.

## Background

The incidence of thyroid cancer increased substantially in the last three decades [1]. Large population-based studies have revealed that differentiated thyroid carcinoma (DTC) contributed to the majority of thyroid cancer, accounting for approximately 90% of all the cases [2]. According to the predominant histologic pattern, DTC is subdivided into papillary thyroid carcinoma (PTC) and follicular thyroid carcinoma (FTC) [3]. PTC is the most frequent and least aggressive subtype of DTC. Among the numerous PTC variants, nearly half of the cases were classical variant papillary thyroid carcinoma (CPTC) and follicular variant papillary thyroid carcinoma (FVPTC) [4]. A recent study demonstrated that FVPTC accounted for up to 41% of all PTC cases [5]. Despite the favorable prognosis of DTC, it has been found that PTC and FTC have distinct clinical behaviors and outcomes; mounting studies demonstrate that FTC has a poorer prognosis [6–8], while some of the studies failed to find the survival difference between PTC and FTC [9]. FVPTC has been considered to possess the features similar to CPTC and FTC because of its follicular architectural pattern and nuclear features of CPTC. FVPTC is historically more aggressive than CPTC, but the clinical reports were controversial. Some previous studies found no significant difference of the clinical outcomes between the two subtypes [10, 11]. While some other studies reported that FVPTC had less aggressive clinical behaviors and similar or even better survival than CPTC [2, 5, 12].

Despite the high morbidity and mortality of cancers in black Americans in the USA, the incidence of thyroid cancer in black Americans is reported to be significantly lower than that in white Americans [13]. However, Brown et al. found that black patients had similar thyroid cancer characteristics and same survival rates as white patients in an equal healthcare system access [14], and a recent study demonstrated that black patients with thyroid cancer had worse outcomes [15]. Moo-Young et al. claimed considerable variability of thyroid cancer across ethnic groups even with the equal healthcare system access [16]. Because of the inconsistent results from these studies, the association between race and survival is undetermined. It is still controversial whether the risk factors for survival are different and whether DTC subtypes have different clinical outcomes in black and white patients. In addition, the association of lymph node and distant metastases with races is to be determined. To answer these questions, we conducted a retrospective analysis using data from the National Cancer Institute’s Surveillance, Epidemiology, and End Results (SEER) Program. The purpose of this study was to determine the risk factors for survival in black and white patients and to compare the survival of DTC subtypes between these two races. Furthermore, the association of races with lymph node and distant metastases was investigated.

## Methods

### Data source and study design

We performed this retrospective cohort analysis using data from SEER database which was designed and maintained by the National Cancer Institute. All patients with CPTC, FVPTC, or FTC diagnosed between 2004 and 2014 were identified using histopathological codes of the International Classification of Disease for Oncology, third edition (ICD-O-3). Non-invasive follicular thyroid neoplasm with papillary-like nuclear features presents extremely low recurrence and metastasis, and it cannot be distinguished from other subtypes of FVPTC due to the limitation of SEER database [17, 18]. Histology codes were listed below to select patients of each subtype accordingly.

CPTC included 8050 (papillary carcinoma not otherwise specified, NOS), 8260 (papillary adenocarcinoma, NOS), and 8343 (papillary carcinoma, encapsulated); FVPTC included 8340 (papillary carcinoma, follicular variant); FTC included 8330 (follicular adenocarcinoma, NOS), 8331 (follicular adenocarcinoma well differentiated), 8332 (follicular adenocarcinoma trabecular), and 8335 (follicular carcinoma, minimal invasive).

Patients whose tumor size, tumor extension, lymph node metastasis, distant metastasis, or removal of lymph nodes were unknown were excluded from this study. Cases without survival times were classified as unknown and were also removed from the study.

Our analysis included demographic variables: sex (male or female), age at diagnosis (< 45 or ≥ 45 years), and race (white or black). Cancer characteristics were classified according to the tumor stage, and tumors were grouped based on the sizes: < 1 cm, 1.1–2 cm, 2.1–4 cm, and > 4 cm. These sizes were chosen according to the American Joint Committee on Cancer T staging. Tumor extension included intrathyroidal extension (codes 100, 200, 300, 400), minimal extrathyroidal extension (code 450), and gross extrathyroidal extension (codes 480, 500, 520, 550, 600, 620, 650, 700, 730, 800); lymph node metastasis (code 000, no lymph nodes metastasis; code 120, metastasis to level VI; codes 135, 155, 158, 160, metastasis to level I, II, III, IV, V, or VII); distant metastasis (code 00, no distant metastasis; code 12, 40, 51, 60, distant metastasis disease). Treatment characteristics included lobectomy, total thyroidectomy, total thyroidectomy with postoperative radioactive iodine (RAI) therapy, and insurance status. All the variables were defined using the SEER specific codes.

Overall survival (OS) and disease-specific survival (DSS) were the two main indexes obtained using the multivariate Cox proportional hazard regressions in our study. Survival time (in months) was calculated for each patient using the “Completed Months of Follow-up” given in the SEER database.

### Statistical analysis

Patients’ demographics and cancer- and treatment-related characteristics were compared between the black and white American groups using chi-square or Fisher’s exact test. For multivariate analysis, Cox proportional hazards regression were used to assess the association between potential risk factors and survival in black and white patients. Adjusted hazard ratios (aHRs) with their respective 95% confidence intervals were used to evaluate the strength of the relative risk between all the factors and survival in different races. HR > 1.0 indicated an increased risk of death. A *P* value < 0.05 was considered statistically significant, and all tests were two-sided. A multivariate logistic regression was used to determine the association of between lymph node and distant metastases in black Americans. All statistical analyses were performed using SPSS 19.0 (IBM Corporation, Armonk, NY).

## Results

### Patient characteristics

Based on the inclusion criteria, a total of 70,346 cases between 2004 and 2014 were eligible for our study. The demographics and clinicopathologic features of DTC were compared between the white and black Americans in Table 1. Among them, 65,171 were white Americans (92.6%), and only 5175 were black Americans (7.4%). The median follow-up time was 50 months for white patients and 46 months for black patients. Of the total death of 2980, 408 died of thyroid cancer. In white patients, 377 died of thyroid cancer (0.6%), and in black patients, 31 died of thyroid cancer (0.6%).

**Table 1:** Patient characteristics within subgroups

Variables	White patients *N* = 65,171(%)	Black patients *N* = 5175(%)	*P* value*
Median follow-up (months)	50	46	
Age at diagnosis (year)			*P* < 0.001
< 45	25,141(38.6)	1936(37.4)	
≥ 45	40,030(61.4)	3239(62.6)	
Sex			*P* < 0.001
Male	15,013(23.0)	867(16.8)	
Female	50,158(77.0)	4308(83.2)	
Histologic type			*P* < 0.001
CPTC	40,383(62.0)	2465(47.6)	
FVPTC	21,138(32.4)	2145(41.4)	
FTC	3650(5.6)	565(10.9)	
Therapy			*P* < 0.001
Lobectomy	8619(13.2)	837(16.2)	
Total thyroidectomy	25,120(38.5)	2223(43.0)	
Total thyroidectomy and radioactive iodine	31,432(48.2)	2115(40.9)	
Removal of lymph nodes			*P* < 0.001
0	31,931(49.0)	3647(70.5)	
1–3	17,412(26.7)	940(18.2)	
≥ 4	15,828(24.3)	588(11.4)	
Stage			*P* < 0.001
I	48,079(73.8)	3810(73.6)	
II	5418(8.3)	538(10.4)	
III	8353(12.8)	646(12.5)	
IV	3321(5.1)	181(3.5)	
Tumor size (mm)			*P* < 0.001
≤ 10	25,593(39.3)	1977(38.2)	
11–20	19,714(30.2)	1203(23.2)	
21–40	14,732(22.6)	1210(23.4)	
> 40	5132(7.9)	785(15.2)	
Median tumor size	13	15	
Tumor extension			*P* < 0.001
Intrathyroidal	55,294(84.8)	4650(89.9)	
Minimal extrathyroidal	4274(6.6)	203(3.9)	
Gross extrathyroidal	5603(8.6)	322(6.2)	
Lymph node metastases			*P* < 0.001
Negative	52,459(80.5)	4708(91.0)	
Level VI	7885(12.1)	284(5.5)	
Level I, II, III, IV, V, or VII	4827(7.4)	183(3.5)	
Distant metastases			0.074
None	64,720(99.3)	5128(99.1)	
Yes	451(0.7)	47(0.9)	
Insurance status			*P* < 0.001
None	1202(1.8)	185(3.6)	
Insured	46,236(70.9)	3319(64.1)	
Any Medicaid	3925(6.0)	617(11.9)	
Unknown	13,808(21.2)	1054(20.4)	
Status			*P* < 0.001
Alive	62,459(95.8)	4907(94.8)	
Dead	2712(4.2)	268(5.2)	
Thyroid cancer	377(0.6)	31(0.6)	
Other	2335(3.6)	237(4.6)	

*CPTC* classical variant papillary thyroid carcinoma, *FVPTC* follicular variant papillary thyroid carcinoma, *FTC* follicular thyroid carcinoma

*P values calculated by Pearson chi-squared or Fisher’s exact testing

White Americans were more likely to have CPTC (62.0%) than black Americans, while black Americans had a higher percentage of FVPTC (41.4%) and FTC (10.9%) than white Americans. Larger tumor (> 4 cm) were more common in black patients (15.2%) than in white patients (7.9%). Lymph node metastasis occurred more frequently in white patients (19.5%) than in black patients (9.0%). Distant metastasis appeared rarely in both white (0.7%) and black patients (0.9%).

### Multivariate analysis

**Fig. 1:**
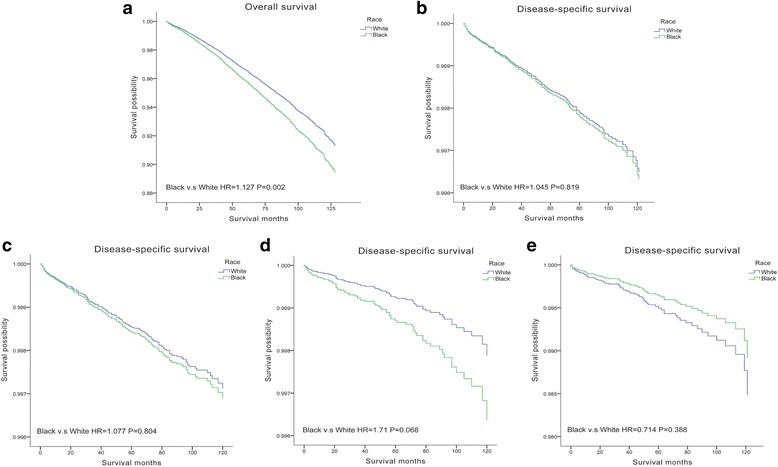
Overall survival (OS) and disease-specific survival (DSS) curves of multivariate Cox analysis. **a** OS is based on race in DTC. **b** DSS is based on race in DTC. **c** DSS is based on race in CPTC. **d** DSS is based on race in FVPTC. **e** DSS is based on race in FTC

Outcomes of OS and DSS were obtained using the multivariate Cox analysis. Black patients had significantly worse OS than white patients (aHR = 1.127, *P* = 0.002), while DSS were comparable between white and black patients (aHR = 1.045, *P* = 0.819). Even when patients are classified into CPTC, FVPTC, and FTC subgroups, no statistical difference of DSS was found between white and black patients **(**Fig. 1).

**Fig. 2:**
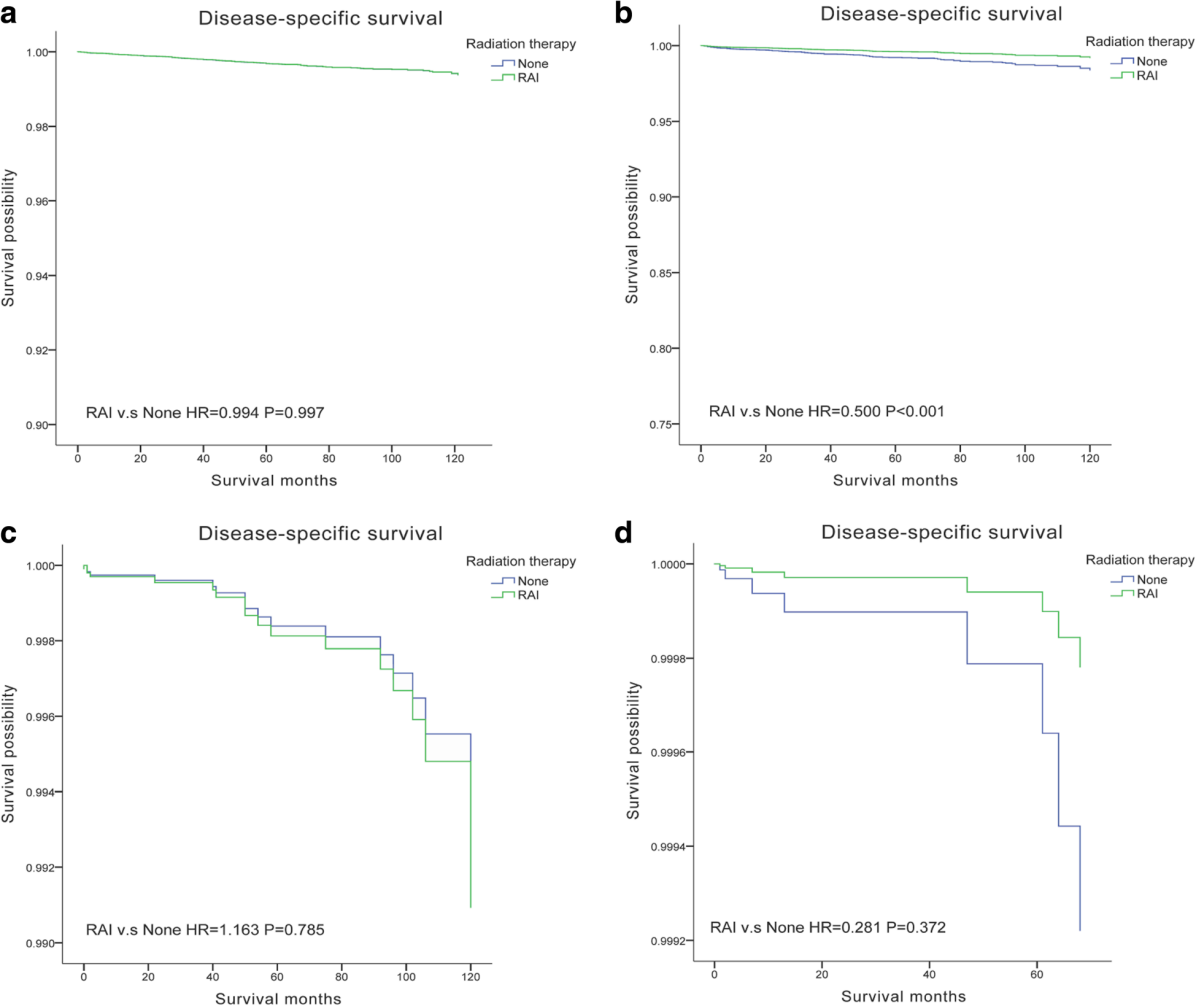
Disease-specific survival (DSS) curves of multivariate Cox analysis. **a** DSS is based on RAI in white patients (tumor > 2 cm without LNM). **b** DSS is based on RAI in white patients (tumor > 2 cm with LNM). **c** DSS is based on RAI in black patients (tumor > 2 cm without LNM). **d** DSS is based on RAI in black patients (tumor > 2 cm with LNM)

Cox proportion hazards models were applied to investigate the association between DSS and potential risk factors in white and black Americans in Table 2. In white Americans, female had better prognosis than male. FVPTC patients had better DSS than CPTC, and FTC patients had significantly worse DSS. Older age (≥ 45 years), advanced stage, larger tumor size, extrathyroidal extension, lymph node metastasis, and distant metastasis had negative effects on DSS in white Americans. For black Americans, despite female had comparable better DSS, no statistical difference was revealed. CPTC, FVPTC, and FTC had very similar DSS. Only tumor size > 4 cm had negative effects on DSS. Minimal extrathyroidal extension and central compartment lymph node metastasis (level VI) did not reveal any significant difference on DSS, while lateral neck nodes (level I, II, III, IV, V, or VII) had negative effects. In addition, black patients did not benefit from a more aggressive therapy than lobectomy, only white patients with total thyroidectomy and postoperative RAI therapy had improved DSS. To further assess the effects of RAI therapy, patients were categorized into three groups: tumor < 2 cm, tumor > 2 cm without lymph node metastasis, and tumor > 2 cm with lymph node metastasis (as RAI was recommended for node-positive and tumor > 2 cm). We found that only white patients with tumor > 2 cm and lymph node metastasis benefited from RAI therapy (Fig. 2). In addition, our results revealed that a number of patients did not receive guideline concordant care (Table 3). As for lymph node dissection, because of the limitation of SEER database, we used the removal of lymph nodes as lymph node surgery. Only black patients with more than four lymph nodes removed had improved DSS.

**Table 2:** Cox proportional hazards regression model analysis of disease-specific survival (DSS) in white and black patients

Variables	White patients		Black patients	
	aHR (95% CI)	*P* value*	aHR (95% CI)	*P* value*
Age at diagnosis (year)				
< 45	Reference		Reference	
≥ 45	6.239(3.338,11.662)	*P* < 0.001	4.489(2.736,8.847)	0.007
Sex				
Male	Reference		Reference	
Female	0.749(0.606,0.927)	0.008	0.627(0.279,1.407)	0.258
Histologic type				
CPTC	Reference		Reference	
FVPTC	0.729(0.555,0.958)	0.023	0.991(0.397,2.472)	0.984
FTC	1.797(1.294,2.498)	*P* < 0.001	0.747(0.224,2.485)	0.634
Therapy				
Lobectomy	Reference		Reference	
Total thyroidectomy	0.779(0.540,1.124)	0.182	0.884(0.255,3.062)	0.846
Total thyroidectomy and radioactive iodine	0.527(0.370,0.750)	*P* < 0.001	0.707(0.209)	2.391
Remove of lymph nodes				
0	Reference		Reference	
1–3	0.870(0.639,1.185)	0.377	2.213(0.798,6.136)	0.127
≥ 4	0.903(0.637,1.280)	0.567	4.107(1.321,12.764)	0.015
Stage				
I	Reference		Reference	
II	1.896(1.083,3.320)	0.025	0.112(0.005,2.455)	0.165
III	1.598(1.018,2.512)	0.041	0.350(0.031,3.974)	0.397
IV	7.799(4.568,13.317)	*P* < 0.001	0.302(0.021,4.404)	0.381
Tumor size (mm)				
≤ 10	Reference		Reference	
11–20	1.102(0.742,1.638)	0.297	0.944(0.140,6.372)	0.953
21–40	1.730(1.157,2.588)	0.008	7.525(0.578,28.028)	0.123
> 40	3.751(2.524,5.574)	*P* < 0.001	21.166(1.746,46.636)	0.017
Tumor extension				
Intrathyroidal	Reference		Reference	
Minimal extrathyroidal	2.137(1.436,3.180)	*P* < 0.001	1.690(0.348,8.205)	0.515
Gross extrathyroidal	3.571(2.630,4.847)	*P* < 0.001	2.938(1.104,7.818)	0.031
Lymph nodes metastases				
Negative	Reference		Reference	
Level VI	2.137(1.436,3.180)	0.017	1.551(0.429,5.600)	0.512
Level I, II, III, IV, V, or VII	1.173(1.01717,1.684)	0.038	2.535(1.604,7.818)	0.035
Distant metastases				
None	Reference		Reference	
Yes	4.961(3.710,6.634)	*P* < 0.001	15.021(3.652,62.452)	*P* < 0.001
Insurance status				
None	Reference		Reference	
Insured	0.680(0.301,1.539)	0.355	0.383(0.045,3.238)	0.378
Any Medicaid	0.621(0.245,1.575)	0.315	2.054(.0235,17.921)	0.515

*aHR* adjusted hazard ratio (adjusted for age at diagnosis, sex, stage, tumor size, extension, lymph node metastases, distant metastases, insurance status and therapy), *CI* confidence interval

*P values calculated by multivariate Cox analysis

**Table 3:** Patient characteristics within subgroups

Variables	White patients *N* = 65,171(%)		Black patients *N* = 5175(%)	
	No RAI	RAI	No RAI	RAI
Tumor size (mm)				
≤ 20	26,645(58.8)	18,662(41.2)	2169(68.2)	1011(31.8)
> 20	7094(35.7)	12,770(64.3)	891(44.7)	1104(55.3)
Lymph node metastases				
Negative	30,670(58.5)	21,789(41.5)	2928(62.6)	1780(37.8)
Positive	3069(24.1)	9643(75.9)	132(28.3)	335(71.7)

To determine whether black patients with DTC associated with higher potential of lymph node and distant metastases, we constructed a multivariate logistic regression model (Table 4). In CPTC and FVPTC patients, black Americans were protective against lymph node metastasis, while for FTC, race was not independently associated with lymph node metastasis. As for distant metastasis, black FVPTC, but not CPTC or FTC, patients had more distant metastasis.

**Table 4:** Multivariate logistic regression model

Variables	Lymph node metastases		Distant metastases	
	OR (95% CI)	*P* value*	OR (95% CI)	*P* value*
CPTC				
White	Reference		Reference	
Black	0.386(0.318,0.437)	*P* < 0.001	1.301(0.756,2.267)	0.373
FVPTC				
White	Reference		Reference	
Black	0.487(0.410,0.563)	*P* < 0.001	1.768(1.137,2.877)	0.023
FTC				
White	Reference		Reference	
Black	0.657(0.316,1.345)	0.278	1.325(0.765,2.412)	0.502

*CPTC* classical variant papillary thyroid carcinoma, *FVPTC* follicular variant papillary thyroid carcinoma, *FTC* follicular thyroid carcinoma, *OR* odds ratio (adjusted for age at diagnosis, sex, tumor size, extension), *CI* confidence interval

*P values calculated by multivariate logistic regression

## Discussion

Thyroid cancer is one of the most common endocrine cancer in the USA, with an estimated incidence of 62,980 cases per year [19]. Racial disparity documentation indicated higher possibility of cancers including colorectal, prostate, lung, and gastric in black Americans [20]. However, the incidence of thyroid cancer is actually lower in black Americans than that in white Americans. The racial differences in the incidence of thyroid cancer has been discussed in many series, while the variations in clinicopathologic characteristics of thyroid cancer and the risk factors in different racial groups are less identified. In our presented study, the DSS of DTC, but not CPTC, FVPTV, or FTC, were almost the same in white and black Americans using multivariate analysis. Race was not an independent risk for death in patients with DTC. It was of note that the overall survival was better in white patients. This might result from the socioeconomic disparities and different lifestyles, such as obesity, cardiovascular disease, misuse of alcohol, and other physical, social, and mental disorders [21]. In the present study, white Americans were more likely to have CPTC, while FVPTC and FTC were more often diagnosed in black Americans. Interestingly, the three subtypes of DTC in white patients had distinct outcomes: DSS of FVPTC was better than CPTC and FTC, and FTC patients had significantly worse prognosis. However, such disparities were not observed in black patients.

In terms of tumor features and behaviors, the larger tumor (> 4 cm) appeared more often in black patients. In contrast to tumor size, extrathyroidal extension and lymph node metastasis occurred more frequently in white patients. Similar results were reported in a previous study that black patients were less likely to present nodal metastasis. In the multivariate logistic regression analysis based on histology, black Americans with CPTC and FVPTC had less lymph node metastasis, while race was not independently associated with lymph node metastasis for FTC. We constructed the same analysis to understand the association between distant metastasis and race. Black Americans with FVPTC, but not CPTC or FTC, had more distant metastasis. These results demonstrated that race might have different impacts on the development of thyroid cancer in different histologic subtypes of DTC.

To further understand the association between race and the potential risk factors, we used Cox proportional hazards models to quantify the clinical significance of patient demographics, tumor characteristics, and treatment characteristics controlling for the remaining variables. It has been well documented that women have a higher incidence of thyroid cancer than men and that men appear to have worse clinical outcomes [22]. Interestingly, the protective effects of female gender were only observed in white Americans based on our results.

The prognostic implications of cervical lymph node metastasis remain controversial. A retrospective analysis by Podnos et al. determined that cervical lymph node metastatic disease was a strong predictor of poor prognosis [23], while other studies failed to identify lymph node metastasis as a risk for death [24]. Lymph node metastases (both central and lateral neck lymph nodes) were significant risk factors for poor outcome in white Americans, while central compartment lymph node metastasis (level VI) did not reveal any statistic decrease of DSS in black Americans. These results compel us to reinvestigate lymph node dissection for patients with DTC. Current guidelines from the American Thyroid Association recommend central compartment (level VI) neck dissection for patients with clinically involved central nodes [25]. Because central compartment lymph node metastasis was not a risk for death in black Americans, the decision of central compartment neck dissection should be further analyzed. Similar results were obtained in tumor extension.

Minimal extrathyroidal extension did not affect the survival of black patients; thus, minimal extrathyroidal extension in this population should not lead to more aggressive surgical or RAI treatment. In our analysis, white patients with total thyroidectomy and postoperative RAI therapy had improved DSS, but not in black patients. Further analysis of RAI therapy revealed that only white patients with tumor > 2 cm and lymph node metastasis could benefit from RAI therapy. In addition, removal of four or more lymph nodes improved the DSS of black patients. These results demonstrated that white and black patients had different risk factors; thus, treatment strategy should be different for the two races.

Several hypotheses have been proposed to explain the disparities between white and black patients with thyroid cancer: socioeconomic status, external sources (provider bias, environmental exposure, lifestyle), and biologic difference [15]. Differences in tumor biology and genetic variances might explain the racial disparities in our study. Black Americans with breast cancer were reported to have differences in progesterone receptor genes [26]. Such differences could also exist in thyroid cancer between white and black Americans. Sex steroid receptor variants and mutations has been found to be associated with cancer development and progression [22]. Thereby, white patients and black patients had different risk factors in our study. Genetic variation among races may contribute to racial disparities, while the potential genes are unclear.

Our study still has several limitations, and the database is potentially biased by prejudice and treatment practices. The SEER database has no information on recurrence due to the rare indolent nature of DTC, but recurrence is more meaningful than death. In addition, the potential genetic differences in white and black patients were not investigated due to the lack of molecular markers included in the SEER database. Lots of confounding factors associated with race were also not considered in our study: alcohol consumption, smoking, education level, income, use of health care, and marital status. These factors may contribute to racial disparities as well.

## Conclusions

Our study demonstrated that the risk factors for DTC survival were significantly different between white and black patients, partially due to genetic variation. Different treatment strategies for thyroid cancer should be considered for different races.

## Abbreviation

CPTC: Classical variant papillary thyroid carcinoma
DSS: Disease-specific survival
DTC: Differentiated thyroid carcinoma
FTC: Follicular thyroid carcinoma
FVPTC: Follicular variant papillary thyroid carcinoma
OS: Overall survival
